# Independent and joint associations of TV viewing time and snack food consumption with the metabolic syndrome and its components; a cross-sectional study in Australian adults

**DOI:** 10.1186/1479-5868-10-96

**Published:** 2013-08-09

**Authors:** Alicia A Thorp, Sarah A McNaughton, Neville Owen, David W Dunstan

**Affiliations:** 1 Baker IDI Heart and Diabetes Institute, Level 4 The Alfred Centre, 99 Commercial Road, Melbourne, Victoria 3004, Australia; 2 The University of Queensland, School of Population Health, Cancer Prevention Research Centre, Brisbane, Level 3, Public Health Building, Herston Road, Herston, Queensland 4006, Australia; 3 Centre for Physical Activity and Nutrition Research, School of Exercise and Nutrition Sciences, Deakin University, 221 Burwood Highway, Burwood, Victoria 3125, Australia; 4 Department of Epidemiology and Preventive Medicine, Monash University, Level 6 The Alfred Centre, 99 Commercial Road, Melbourne, Victoria 3004, Australia; 5 Department of Medicine, Monash University, Bld 15, Wellington Road, Clayton, Victoria 3800, Australia; 6 School of Population Health, The University of Melbourne, 207 Bouverie St, Parkville 3010, Victoria 3010, Australia; 7 School of Sport Science, Exercise and Health, The University of Western Australia, 35 Stirling Highway, Crawley, Perth 6009, Australia

**Keywords:** Snacking, Sedentary behaviour, Screen-time, Metabolic risk

## Abstract

**Background:**

Television (TV) viewing time is positively associated with the metabolic syndrome (MetS) in adults. However, the mechanisms through which TV viewing time is associated with MetS risk remain unclear. There is evidence that the consumption of energy-dense, nutrient poor snack foods increases during TV viewing time among adults, suggesting that these behaviors may jointly contribute towards MetS risk. While the association between TV viewing time and the MetS has previously been shown to be independent of adult’s overall dietary intake, the specific influence of snack food consumption on the relationship is yet to be investigated. The purpose of this study was to examine the independent and joint associations of daily TV viewing time and snack food consumption with the MetS and its components in a sample of Australian adults.

**Methods:**

Population-based, cross-sectional study of 3,110 women and 2,572 men (>35 years) without diabetes or cardiovascular disease. Participants were recruited between May 1999 and Dec 2000 in the six states and the Northern Territory of Australia. Participants were categorised according to self-reported TV viewing time (low: 0-2 hr/d; high: >2 hr/d) and/or consumption of snack foods (low: 0-3 serves/d; high: >3 serves/d). Multivariate odds ratios [95% CI] for the MetS and its components were estimated using gender-specific, forced entry logistic regression.

**Results:**

OR [95% CI] for the MetS was 3.59 [2.25, 5.74] (p≤0.001) in women and 1.45 [1.02, 3.45] (p = 0.04) in men who jointly reported high TV viewing time and high snack food consumption. Obesity, insulin resistance and hypertension (women only) were also jointly associated with high TV viewing time and high snack food consumption. Further adjustment for diet quality and central adiposity maintained the associations in women. High snack food consumption was also shown to be independently associated with MetS risk [OR: 1.94 (95% CI: 1.45, 2.60), p < 0.001] and hypertension [OR: 1.43 (95% CI: 1.01, 2.02), p = 0.05] in women only. For both men and women, high TV viewing time was independently associated with the MetS and its individual components (except hypertension).

**Conclusion:**

TV viewing time and snack food consumption are independently and jointly associated with the MetS and its components, particularly in women. In addition to physical activity, population strategies targeting MetS prevention should address high TV time and excessive snack food intake.

## Background

While certain lifestyle behaviors such as physical activity [1] and diet [2] have been shown to be inversely associated with the metabolic syndrome (MetS), recent evidence from cross-sectional studies have also identified time spent in leisure-time sedentary behaviors [3-7] and overall sitting time [8,9] to be independently associated with MetS risk in adults. Sedentary behaviors are a distinct class of behaviors that are characterised primarily by prolonged sitting [10]. Time spent watching television (TV), one of the most common sedentary behaviors of adults during leisure time, has been shown to be positively associated with the presence of the MetS and with elevated risk scores for the MetS independent of leisure-time physical activity, diet and socio-demographic factors [3-5], with the associations being stronger in women.

Although the mechanisms through which TV viewing time increases MetS risk remain unclear, one lifestyle behavior proposed as a potential mediator is poor dietary habits, specifically the consumption of energy-dense, nutrient poor snack foods [11]. Population-based studies have reported that TV viewing time is associated with an unhealthy dietary pattern in adults. Specifically, high levels of TV viewing time are associated with a “convenience food pattern” in men [12] and higher intakes of energy-dense snack foods in women [13]. In experimental studies, snacking frequency and the consumption of energy-dense, nutrient poor snack foods and beverages have been found to increase during TV viewing time among adults [14]. It has been suggested that this may be a consequence of exposure to snack-food advertising during TV viewing, which increases adult’s propensity to consume such foods [15].

While the detrimental association between TV viewing time and the MetS in adults has been shown to be independent of overall dietary intake [3], the direct influence of energy-dense, nutrient poor snack food consumption is yet to be established. Cross-sectional studies have shown that food and beverage consumption during TV viewing partially mediates the association of TV viewing time with components of the MetS such as abdominal obesity [11] and body weight status [14], suggesting that snack food consumption may have a similar influence on the relationship between TV viewing time and the MetS.

In a large population-based sample of Australian adults, we examined the independent and joint associations of daily TV viewing time and snack food consumption with the MetS and its components.

## Methods

### Participants

The study population consisted of participants from the baseline cohort of the Australian Diabetes, Obesity and Lifestyle study (AusDiab); a, population-based prospective study of national diabetes mellitus incidence and associated risk factors. Non-institutionalised adults aged ≥ 25 years without physical or intellectual disabilities were invited to attend an initial household interview followed by a biomedical examination (which included an oral glucose tolerance test). Data was collected over a 21 month period between May 1999 and December 2000 in the six states and the Northern Territory of Australia from a total of 42 randomly selected urban and nonurban areas based on Census Collector Districts. The study methods, response rates, sample representativeness and main findings have been reported in detail elsewhere [16-18].

Briefly, 28,033 households were approached to participate in the study from the 42 selected clusters. Of the 19, 215 households successfully contacted, 17,129 were deemed eligible and 11, 249 agreed to participate in the initial household interview. A total of 20,347 eligible adults completed the initial household interview and 11, 247 (55.3%) attended the follow-up biomedical examination. From the 11,247 study participants, we further excluded those who were aged ≤ 35 years for whom fasting insulin data was not available (n = 1603), pregnant (n = 60), had been clinically diagnosed with diabetes (n = 475), had a history of cardiovascular disease (n = 938), reported taking anti-hypertensive or lipid-lowering medication (n = 2727), reported an implausible TV viewing time (>18 hr on a weekday or weekend day; n = 93), did not complete a food frequency questionnaire (FFQ; n = 204), reported an implausible dietary intake according to established criteria [19] (n = 582), had an invalid FFQ due to missing dietary data (>10% of FFQ; n = 476), or whose MetS status could not be ascertained due to missing data (n = 449). Exclusion criteria were not mutually exclusive, so participants could be excluded based on more than one criterion. There was no marked difference in the gender ratio of included and excluded participants (0.83 vs 0.80). However, those included were for the most part older, had lower BMI and had completed further education than those excluded. The final study sample consisted of 3,110 women and 2,572 men.

### Assessment of metabolic syndrome components

Participants who attended the follow-up biomedical examination provided a fasting blood sample and underwent a 75-g oral glucose tolerance test at a local survey centre after an overnight fast (>8 hr). Plasma glucose levels (fasting and 2-hr post-load) were determined by a spectrophotometric-hexokinase method; fasting serum triglycerides and HDL-cholesterol were measured by enzymatic methods (Roche Modular, Roche Diagnostics, Indianapolis, USA) on an Olympus AU600 analyzer (Olympus Optical, Tokyo, Japan). Fasting serum insulin levels were determined using a human insulin-specific radioimmunoassay kit (Linco Research, St. Charles, MO). Microalbuminuria levels were determined using an immunoturidimetric method (Olympus AU600 analyzer). Height, weight, duplicate waist circumference and triplicate resting blood pressure measurements were conducted by trained personnel according to methods that have been previously published [17].

### Diagnosis of the metabolic syndrome

Clinical criteria for the diagnosis of the MetS and its individual components was based on the 1999 WHO working definition [20] which includes the presence of either diabetes (fasting plasma glucose ≥7.0 mmol/L or 2-hr plasma glucose ≥ 11.1 mmol/L), impaired glucose tolerance (fasting plasma glucose <7.0 mmol/L and 2-hr plasma glucose ≥ 7.8 but <11.1 mmol/L) or insulin resistance (fasting insulin levels in the upper quartile of the non-diabetic population ) following a glucose tolerance test, plus two or more of the following; obesity (defined as a body mass index >30 kg/m^2^ or waist:hip ratio >0.9 for men or >0.85 for women), dyslipidemia (defined as triglycerides ≥ 1.7 mmol/L or HDL-cholesterol <0.9 mmol/L for men or <1.0 mmol/L for women), hypertension (defined as blood pressure ≥ 140/90 mm Hg) and microalbuminuria (defined as urinary albumin excretion ≥ 20 μg/min).

For comparative purposes, clinical criteria for the diagnosis of the MetS based on the harmonized definition provided by the International Diabetes Federation Task Force on Epidemiology and Prevention; Nation Heart, Lung, and Blood Institute; American Heart Association; World Heart Federation; International Atherosclerosis Society; and International Association for the Study of Obesity was also analysed [21].

### Assessment of TV viewing time

Self-reported TV viewing time was determined by asking participants to recall how much time they spent watching TV or DVDs, separately across all work days and non-work days during the preceding seven days. The recall measure only included time when TV viewing was the main activity and not when the TV was just switched on or when other activities were being undertaken concurrently. TV viewing time was then calculated using the following formula [(workdays TV viewing + non workdays TV viewing / 7] to determine hours per day spent watching TV. This measure has previously been shown to provide a reliable (intraclass correlation = 0.82 (95% CI: 0.75, 0.87) and valid (criterion validity = 0.3) estimate of TV viewing time in adults [22]. Based on previously identified associations with individual components of MetS risk [3], daily TV viewing time (hr/d) was categorised into two groups: 0–2 hr/d (low) or >2 hr/d (high).

### Assessment of snack food consumption

Daily consumption of snack foods was assessed using a self-administered, food frequency questionnaire (FFQ) developed by the Victorian Anti-Cancer Council of Victoria for use in Australian adults [23]. The 74-item FFQ has previously been validated against a 7-day weighed food diary with correlation coefficients for energy-adjusted nutrient intakes in the range of 0.28 (vitamin A) to 0.78 (carbohydrate) [23].The present analyses are based on a subset of nine food items from the FFQ that represent common snack foods: crackers, sweet biscuits, cakes/pastries, meat pies/pastries/quiches or other, chocolate, flavoured milk, potato chips/crisps, ice cream, hot (fried) chips. Food items were selected according to the Australian Guide to Healthy Eating (AGHE) guidelines for “non-core” foods which are representative of common snack foods available today. Non-core foods are defined as foods that are not essential to provide nutrient requirements and contain too much fat, sugar and salt and are low in dietary fibre [24].

Participants reported how frequently they consumed the nine snack food items during the previous month, using the following seven response categories: never or not in the last month, several times per month, once a week, a few times a week, on most days, once per day and several times per day. Daily snack food consumption (number of serves per day) was determined using an established method of converting the frequency of consumption during the previous month for each food item into a daily equivalent score [19]. To control for variation in the serving size of the nine food items, a gender-specific portion size calibrator was derived for each participant based on their individual responses to photos of scaled portions of four foods in the FFQ. Daily equivalent scores for each of the nine food items were then multiplied by the portion size calibrator and the resultant scores combined to calculate daily consumption of snack foods (serves/d).

Consumption of snack foods was categorised into two groups based on self-reported daily intake; 0–3 serves of snack foods per day (low) and >3 serves of snack foods per day (high). These pragmatic cuff-off values for the two categories were partly informed by the current AGHE gender and age-group specific recommendations for the consumption of non-core foods [24]. According to the AGHE, the recommended daily consumption of non-core foods is up to 2.5 serves for women and up to 3 serves for men if aged 19–60 years.

### Assessment of demographic and behavioral attributes

Demographic (age, gender, parental history of diabetes, education level, employment status) and behavioral attributes (leisure-time physical activity, smoking status, daily alcohol intake, diet quality) of participants were assessed using interviewer-administered questionnaires.

Leisure-time physical activity was assessed using the Active Australia Survey Questionnaire [25] which has been shown to provide a reliable (intraclass correlation = 0.59 (95% CI: 0.52, 0.65) and valid (criterion validity = 0.3) estimate of leisure-time physical activity amongst Australian adults [26,27]; total leisure-time physical activity (min/wk) was calculated by methods previously described [28].

Self-reported FFQ data was used to calculate daily alcohol intake (KJ/d) and to derive a measure of diet quality (the Dietary Guideline Index) based on adherence to Australian dietary guidelines for the consumption of five “core” food groups (vegetables, fruits, cereals, meat and alternatives, dairy) and “non-core” foods (i.e. energy-dense, nutrient poor foods) (Diet Guideline Index –scale 1–130 with 130 representing high diet quality) [29,30].

### Ethics

The Ethics Committee of the Baker IDI Heart and Diabetes Institute (formerly known as the International Diabetes Institute) approved the study, and written informed consent was obtained from all participants.

### Statistical analysis

Statistical analyses were conducted using STATA Statistical Software Package, Release 11.0 (STAT, College Station, TX, USA) survey commands for analysing complex survey data. Sample weights based on the 1998 estimated residential Australian population aged >35 years were used to account for clustering and stratification in the survey design and for non-response. Data were stratified according to gender, due to a statistically significant interaction effect between gender and MetS risk (results not shown). Unadjusted means with 95% confidence intervals were calculated for continuous confounders (age, leisure-time physical activity, total alcohol intake and diet quality) and percentages with 95% confidence intervals for categorical confounders (education level, smoking status, employment status, parental history of diabetes). Regression analyses were used to determine gender-specific differences in potential confounders across joint categories of TV viewing time and snack food consumption. Bivariate correlations (Spearman’s r) assessed the gender-specific relationships between exposure variables, snack food consumption (serves/d) and TV viewing time (hr/d). Continuous measures of TV viewing time and snack food consumption were presented as geometric means with 95% confidence intervals. Odds ratios (with 95% confidence intervals) for the MetS and for individual components of the MetS according to separate categories of snack food consumption and TV viewing time were estimate using gender-specific, forced entry logistic regression models. Model A adjusted for age (years) only. Model B additionally adjusted for education level (completed university or higher education/no further education), smoking status (current smoker/non-smoker), employment status (employed/unemployed), parental history of diabetes (yes/no), leisure-time physical activity (min/wk) and daily alcohol intake (KJ/d). To examine the independent associations of TV viewing time (hr/d) and snack food consumption (serves/d), with risk for the MetS and its components, Model B was adjusted by each variable accordingly (Model C). To further control for the confounding effect of diet, Model C was additionally adjusted for diet quality (Model D). For individual components of the MetS (excluding the measure of obesity), a fifth model (Model E) additionally adjusted for waist circumference.

To test the joint association of TV viewing time and snack food consumption with risk for the MetS and its individual components, gender-specific, forced-entry logistic regression models that combined categories of TV viewing time and snack food consumption were created. The categories included: 0–2 hr/d of TV viewing and 0–3 serves/d of snack foods (reference category, low TV/low snack food), 0–2 hr/d of TV viewing and >3 serves/d of snack foods(low TV/high snack food), >2 hr/d of TV viewing and 0–3 serves/d of snack foods (high TV/low snack food ), >2 hr/d of TV viewing and >3 serves/d of snack foods (high TV/ high snack food). Models adjusted for age (Model A) and potential confounders including educational level, smoking status, employment status, parental history of diabetes, leisure-time physical activity and daily alcohol intake (Model B). Model C additionally adjusted for diet quality. For individual components of the MetS (excluding the measure of obesity), a forth model (Model D) additionally adjusted for waist circumference. For all statistical tests, a p-value ≤ 0.05 was considered significant. As there were no missing values for any exposure, outcome or confounder measures, the sample size is the same for all analyses.

## Results

Of the 5,682 participants included in the analyses, 168 (5.4%) women and 319 (12.4%) men were classified as having the MetS according to WHO criteria. Table 1 shows selected characteristics for men and women according to combined categories of TV viewing time and snack food consumption. With the exception of leisure-time physical activity, parental history of diabetes, daily alcohol intake and diet quality (women only), significant differences were observed across the four categories for the socio-demographic, behavioral and anthropometric measures in both men and in women.

**Table 1:** Characteristics of study participants according to combined categories of TV viewing time and snack food consumption

	**0-2 hr/d TV** &**0–3 snacks/d**	**0-2 hr/d TV** &**>3 snacks/d**	**>2 hr/d TV** &**0–3 snacks/d**	**>2 hr/d TV** &**>3 snacks/d**	**P**
N=					
M	1,167	482	604	319	-
W	1,870	355	717	168	-
Age (years)					
M	50.3 (49.2, 51.3)	47.9 (46.7, 49.2)	53.3 (51.4, 55.3)	50.0 (47.8, 52.3)	0.02
W	48.7 (47.7, 49.6)	48.2 (46.4, 50.1)	55.3 (53.5, 57.2)	53.3 (49.4, 57.1)	>0.001
Parental History of Diabetes (%)					
M	19.6 (16.3, 23.0)	12.9 (8.5, 17.4)	19.9 (15.4, 24.4)	16.5 (10.5, 22.4)	0.52
W	18.0 (15.6, 20.4)	17.0 (13.0, 21.1)	17.9 (13.7, 22.2)	18.5 (10.9, 26.2)	0.98
Current Smoker (%)					
M	15.5 (12.2, 18.8)	13.0 (7.6, 18.5)	22.1 (17.2, 26.9)	19.6 (10.9, 28.3)	0.001
W	10.7 (8.1, 13.4)	10.4 (6.9, 13.8)	16.5 (11.5, 21.4)	20.8 (11.1, 30.4)	0.02
Completed University or Higher Education (%)					
M	54.4 (48.5, 60.3)	50.0 (41.0, 59.0)	40.7 (33.8, 47.5)	43.6 (34.2, 53.0)	0.001
W	45.4 ( 40.3, 50.5)	44.6 (35.4, 53.8)	23.4 (18.1, 28.8)	13.8 (6.1, 21.4)	>0.001
Employed (%)					
M	80.4 (77.1, 83.8)	86.3 (81.9, 90.7)	61.8 (53.9, 69.7)	75.9 (65.7, 86.1)	0.01
W	69.6 (65.2, 74.0)	67.1 (59.8, 74.5)	42.0 (36.2, 47.7)	42.5 (27.3, 57.7)	>0.001
BMI (kg/m^2^)					
M	26.5 (26.0, 26.9)	26.4 (26.2, 26.7)	27.4 (27.0, 27.8)	27.3 (26.6, 28.0)	>0.001
W	25.7 (25.3, 26.1)	25.9 (25.0, 26.9)	27.3 (26.5, 28.0)	27.5 (25.6, 29.4)	>0.001
Waist (cm)					
M	95.0 (93.3, 96.6)	94.8 (93.9, 95.7)	98.4 (96.9, 99.9)	98.1 (96.5, 99.7)	>0.001
W	81.8 (80.3, 83.4)	82.9 (80.5, 85.3)	86.2 (84.5, 88.0)	86.3 (81.6, 90.9)	>0.001
Diet Quality Index					
M		82.5 (81.2, 83.8)	79.5 (78.0, 81.0)	79.8 (77.6, 82.0)	77.7 (75.4, 79.9)	>0.001
W	86.8 (86.0, 87.6)	83.8 (82.2, 85.4)	87.3 (85.0, 89.5)	85.4 (80.8, 90.0)	0.56	
Daily Alcohol Intake (KJ/d)						
M	685 (603, 768)	586 (489, 683)	652 (558, 746)	599 (498, 701)	0.10	
W	295 (254, 336)	217 (178, 256)	276 (220, 331)	284 (200, 369)	0.27	
Leisure-Time Physical Activity (min/wk)						
M	319 (285, 352)	354 (291, 417)	311 (252, 371)	321 (264, 378)	0.94	
W	230 (197, 263)	221 (168, 274)	209 (178, 240)	218 (164, 272)	0.43	

Data are weighted to the Australian population and are presented as unadjusted means with 95% confidence intervals in parentheses or as percentages with 95% confidence intervals in parentheses.

Significant gender differences in daily TV viewing time (men: 1.42 (95% CI: 1.32, 1.52); women: 1.20 (95% CI: 1.12, 1.28), p < 0.001) and snack food consumption (men: 2.56 (95% CI: 2.40, 2.73); women: 1.67 (95% CI: 1.58, 1.77), p < 0.001) were observed. There was also a weak, albeit significant positive relationship between consumption of snack foods (serves/d) and TV viewing time (hr/d) reported for men (r = 0.07, p = 0.0007) and for women (r = 0.05, p = 0.005).

Table 2 describes the extent to which TV viewing time and snack food consumption independently contribute to MetS risk in adults. For both men and women, high TV viewing time (>2 hr/d) was associated with a greater odds for the MetS, independent of snack food consumption. In women only, high snack food consumption (>3 serves/d) was shown to be associated with a greater odds for the MetS, independent of TV viewing time. Further adjustment for diet quality maintained all significant relationships observed in both men and in women.

**Table 2:** Age- and multivariate adjusted odds ratio for presence of the MetS according to separate categories of TV viewing time (>2 hours/day) and snack food consumption (>serves/day) in Australian men and women

	**Adjusted OR (95%CI) for MS based on WHO criteria**			
	Men	P-value	Women	P-value
TV viewing time (> 2 hr/d)				
Model A	1.47 (1.04, 2.08)	0.03	1.89 (1.33, 2.70)	0.001
Model B	1.46 (1.07, 2.01)	0.02	1.75 (1.20, 2.54)	0.004
Model C	1.47 (1.07, 2.02)	0.02	1.72 (1.18, 2.50)	0.006
Model D	1.43 (1.04, 1.98)	0.03	1.72 (1.19, 2.50)	0.005
Snack Food Consumption ( > 3 serves/d)				
Model A	0.86 (0.61, 1.23)	0.40	2.13 (1.60, 2.84)	>0.001
Model B	0.91 (0.63, 1.31)	0.59	2.04 (1.53, 2.73)	>0.001
Model C	0.88 (0.61, 1.29)	0.52	1.94 (1.47, 2.58)	>0.001
Model D	0.84 (0.57, 1.25)	0.38	1.94 (1.45, 2.60)	>0.001

Data presented as adjusted odds ratios with 95% confidence intervals in parentheses.

MetS status based on 1999 WHO criteria.

Model A: adjusted for age (years) only.

Model B: adjusted for age (years), education level (completed university or higher education/no further education), smoking status (current smoker/ non-smoker), employment status (employed/unemployed), parental history of diabetes (yes/no), leisure-time physical activity (mins/wk), and daily alcohol intake (KJ/d).

Model C: adjusted for Model B + snack food consumption (serves/d) or TV viewing time (hr/d).

Model D: adjusted for Model C + diet quality.

For TV viewing time only, similar independent associations were observed in men and in women when using the harmonized definition for the MetS (data not shown). No independent associations were observed in relation to snack food consumption.

Consistent with our previous findings [3], in both men and in women, high TV viewing time was associated with the presence of dyslipidaemia, diabetes (or impaired glucose tolerance/impaired fasting glucose), obesity and insulin resistance independent of snack food consumption and diet quality (Model D; data not shown). Further adjustment for waist circumference, maintained all associations reported in women.

With the exclusion of hypertension which was positively associated with high snack food consumption in women independent of TV viewing time and diet quality [OR: 1.43 (95% CI: 1.01, 2.02), p = 0.05); Model D], but not waist circumference (p = 0.07), no significant multivariate associations were observed between high snack food consumption and individual components of the MetS in men or in women.

**Figure 1:**
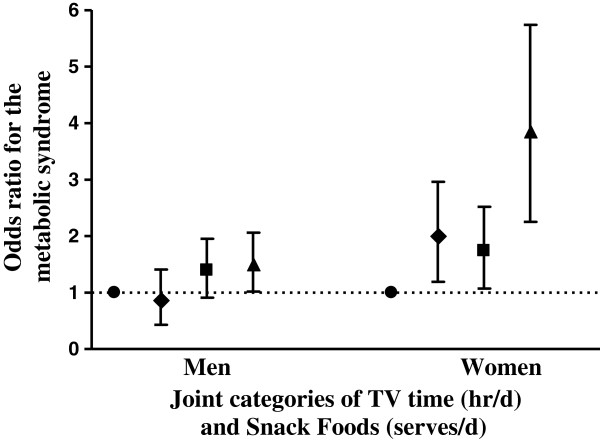
**Multivariate adjusted odds ratios (95%CI) for the presence of the metabolic syndrome according to joint categories of TV viewing time and snack food consumption in Australian men and woman.** Low TV time/Low snack food consumption (0-2 hr/d TV & 0-3 snacks/d): *closed circles* (*reference category*); Low TV time/High snack food consumption (0-2 hr/d TV & >3 snacks/d): *closed diamond*; High TV time/Low snack food consumption (>2 hr/d TV & 0-3 snacks/d): closed squares; High TV time/High snack food consumption (>2 hr/d TV & >3 snacks/d): *closed triangles*.

As illustrated in the Figure 1, the multivariate adjusted OR for the MetS was 3.59 [95% CI: 2.25, 5.74] (p < 0.001) for women in the high TV time/high snack food category relative to the reference category: low TV time/low snack food. There was also a significant increased risk for the MetS in women with low TV/high snack food (OR: 1.88 [95% CI: 1.19, 2.96], p = 0.008) and high TV/low snack food (OR: 1.64 [95% CI: 1.07, 2.52], p = 0.03) compared to those in the reference category. In men, only those in the high TV/high snack food category were at increased risk for the MetS (OR: 1.45 [95% CI: 1.02, 3.45], p = 0.04). Further adjustment for diet quality maintained the significant joint associations with the MetS in women but not in men.

Multivariate analyses according to the harmonised criteria for the MetS revealed a significant, albeit attenuated joint association between high TV time/high snack food and risk for the MetS in women, but did not reach statistical significance in men (p = 0.06). Compared to women in the low TV/low snack food category, the ORs for the MetS was 1.88 [95% CI: 1.02, 3.45] (p = 0.04) for those in the high TV/high snack food category. Further adjustment of diet quality did not alter the observed association.

For both men and women, TV viewing time and snack food consumption were jointly associated with individual components of the MetS (Table 3). Adults in the high TV time/ high snack food category were more likely to be obese, have insulin resistance and have hypertension (women only), compared to those in the low TV time/ low snack food category (Table 3, Model B). With the exception of insulin resistance (men only), further adjustment for diet quality and waist circumference maintained the joint association between high TV time/ high snack food and components of the MetS.

**Table 3:** Odds ratios for the presence of MetS components (WHO criteria) according to joint categories of TV viewing time and snack food consumption in Australian men and women

		**Adjusted OR (95%CI) for MetS Components based on WHO criteria**						
		0-2 hr/d TV & 0–3 snacks/d	0-2 hr/d TV & >3 snacks/d	P	>2 hr/d TV & 0–3 snacks/d	P	>2 hr/d TV & >3 snacks/d	P
N=								
	M	1,167	482		604		319	
	W	1,870	355		717		168	
Insulin Resistance								
Model A	M	1.00	0.73 (0.48, 1.11)	0.14	1.41 (0.99, 2.00)	0.05	1.51 (0.99, 2.30)	0.05
	W	1.00	1.27 (0.84, 1.91)	0.25	1.88 (1.38, 2.55)	>0.001	2.15 (1.35, 3.44)	0.002
Model B	M	1.00	0.75 (0.48, 1.16)	0.19	1.45 (1.04, 2.01)	0.03	1.54 (1.02, 2.34)	0.04
	W	1.00	1.18 (0.76, 1.83)	0.45	1.82 (1.26, 2.64)	0.002	2.14 (1.44, 3.18)	>0.001
Model C	M	1.00	0.70 (0.45, 1.09)	0.11	1.39 (1.01, 1.92)	0.05	1.42 (0.93, 2.18)	0.11
	W	1.00	1.11 (0.72, 1.70)	0.63	1.83 (1.26, 2.65)	0.002	2.11 (1.36, 3.29)	0.002
Model D	M	1.00	0.68 (0.43, 1.06)	0.08	1.02 (0.78, 1.33)	0.89	1.09 (0.64, 1.85)	0.74
	W	1.00	0.97 (0.60, 1.55)	0.88	1.60 (1.10, 2.32)	0.02	1.82 (1.25, 2.65)	0.003
Diabetes/IGT/IFG								
Model A	M	1.00	0.76 (0.49, 1.18)	0.22	1.31 (0.93, 1.85)	0.12	1.37 (0.93, 2.02)	0.11
	W	1.00	1.13 (0.74, 1.73)	0.57	1.61 (1.18, 2.19)	0.004	2.34 (1.51, 3.63)	>0.001
Model B	M	1.00	0.85 (0.51, 1.41)	0.53	1.10 (0.75, 1.60)	0.62	1.30 (0.82, 2.04)	0.25
	W	1.00	0.97 (0.60, 1.57)	0.91	1.13 (0.81, 1.58)	0.46	1.68, 0.81, 3.50)	0.16
Model C	M	1.00	0.82 (0.50, 1.34)	0.42	1.07 (0.73, 1.58)	0.72	1.23 (0.78, 1.94)	0.37
	W	1.00	0.95 (0.58, 1.56)	0.83	1.14 (0.82, 1.58)	0.43	1.67 (0.82, 3.41)	0.16
Model D	M	1.00	0.83 (0.51, 1.34)	0.42	0.92 (0.63, 1.35)	0.66	1.07 (0.67, 1.71)	0.78
	W	1.00	0.91 (0.55, 1.50)	0.70	1.02 (0.72, 1.43)	0.92	1.54 (0.60, 3.97)	0.36
Obesity								
Model A	M	1.00	0.90 (0.61, 1.31)	0.56	1.49 (1.10, 2.01)	0.01	1.70 (1.23, 2.34)	0.002
	W	1.00	1.23 (0.86, 1.76)	0.25	1.69 (1.18, 2.41)	0.01	2.48 (1.22, 5.04)	0.01
Model B	M	1.00	0.92 (0.62, 1.37)	0.68	1.45 (1.10, 1.92)	0.01	1.68 (1.20, 2.34)	0.003
	W	1.00	1.19 (0.82, 1.74)	0.35	1.54 (1.05, 2.25)	0.03	2.18 (1.03, 4.62)	0.04
Model C	M	1.00	0.87 (0.58, 1.31)	0.50	1.40 (1.06, 1.85)	0.02	1.57 (1.11, 2.23)	0.01
	W	1.00	1.21 (0.83, 1.76)	0.31	1.53 (1.05, 2.25)	0.03	2.19 (1.05, 4.57)	0.04
Hypertension								
Model A	M	1.00	0.89 (0.65, 1.22)	0.45	1.15 (0.78, 1.70)	0.47	1.26 (0.76, 2.10)	0.36
	W	1.00	1.16 (0.70, 1.92)	0.56	1.26 (0.96, 1.65)	0.10	2.34 (1.21, 4.53)	0.01
Model B	M	1.00	0.95 (0.71, 1.26)	0.70	1.15 (0.78, 1.69)	0.48	1.32 (0.78, 2.23)	0.29
	W	1.00	1.24 (0.76, 2.01)	0.38	1.26 (0.92, 1.72)	0.15	2.32 (1.17, 4.59)	0.02
Model C	M	1.00	0.90 (0.67, 1.23)	0.51	1.12 (0.77, 1.62)	0.56	1.24 (0.72, 2.12)	0.43
	W	1.00	1.23 (0.75, 2.01)	0.41	1.26 (0.92, 1.73)	0.15	2.32 (1.18, 4.55)	0.02
Model D	M	1.00	0.92 (0.67, 1.25)	0.57	1.00 (0.70, 1.42)	0.98	1.11 (0.65, 1.90)	0.70
	W	1.00	1.20 (0.73, 1.98)	0.47	1.17 (0.85, 1.60)	0.32	2.24 (1.00, 5.00)	0.05
Dyslipidaemia								
Model A	M	1.00	0.84 (0.60,1.17)	0.29	1.31 (1.03, 1.67)	0.03	1.14 (0.77, 1.69)	0.51
	W	1.00	1.23 (0.71,2.13)	0.45	2.01 (1.44,2.80)	>0.001	1.90 (0.99, 3.63)	0.05
Model B	M	1.00	0.90 (0.63, 1.28)	0.55	1.33 (1.06, 1.67)	0.02	1.17 (0.78, 1.77)	0.43
	W	1.00	1.20 (0.68, 2.13)	0.51	1.85 (1.33, 2.59)	0.001	1.69 (0.85, 3.38)	0.13
Model C	M	1.00	0.87 (0.63, 1.22)	0.41	1.31 (1.05, 1.64)	0.02	1.13 (0.78, 1.65)	0.50
	W	1.00	1.23 (0.69, 2.16)	0.47	1.85 (1.32, 2.59)	0.001	1.70 (0.85, 3.38)	0.13
Model D	M	1.00	0.86 (0.62, 1.21)	0.38	1.10 (0.88, 1.39)	0.37	0.96 (0.61, 1.49)	0.85
	W	1.00	1.15 (0.65, 2.03)	0.63	1.65 (1.21, 2.24)	0.002	1.46 (0.75, 2.83)	0.26
Microalbuminuria								
Model A		1.00	1.01 (0.39, 2.61)	0.98	0.80 (0.43, 1.48)	0.46	1.26 (0.65, 2.43)	0.48
		1.00	1.14 (0.48, 2.73)	0.76	1.09 (0.57, 2.09)	0.79	2.97 (1.04, 8.48)	0.04
Model B	M	1.00	1.08 (0.48, 2.80)	0.87	0.68 (0.36, 1.28)	0.23	1.26 (0.60, 2.68)	0.53
	W	1.00	1.04 (0.30, 3.60)	0.95	0.85 (0.26, 2.78)	0.79	0.31 (0.04, 2.33)	0.25
Model C	M	1.00	0.53 (0.20, 1.46)	0.22	0.86 (0.45, 1.65)	0.65	0.78 (0.32, 1.89)	0.57
	W	1.00	0.94 (0.27, 3.24)	0.92	0.87 (0.28, 2.72)	0.80	0.29 (0.04, 2.25)	0.23
Model D	M	1.00	0.53 (0.19, 1.47)	0.22	0.83 (0.45, 1.54)	0.55	0.75 (0.30, 1.85)	0.52
	W	1.00	0.94 (0.28, 3.15)	0.92	0.85 (0.27, 2.72)	0.78	0.28 (0.04, 1.98)	0.19

Data presented as adjusted odds ratio with 95% confidence intervals in parentheses.

Model A: adjusted for age (years) only.

Model B: adjusted for age (years), education level (completed university or higher education/no further education), smoking status (current smoker/ non-smoker), employment status (employed/unemployed), parental history of diabetes (yes/no), leisure-time physical activity (mins/wk) and daily alcohol intake (KJ/d).

Model C: adjusted for Model B + diet quality.

Model D: adjusted for Model C + waist circumference.

IGT = impaired glucose tolerance; IFG = impaired fasting glucose.

Engaging in high TV viewing time in the presence of low snack food consumption was also shown to increase risk for obesity, insulin resistance and dyslipidemia in men and women, compared to those in the low TV time/low snack food category (Table 3, Model B). In women, the associations were independent of diet quality and waist circumference; in men, waist circumference was shown to significantly attenuate the associations observed.

## Discussion

In this large, population-based study, we found that TV viewing time and snack food consumption were jointly associated with the MetS and its components in Australian adults. Specifically, men and women who engaged in both high TV viewing time and high snack food consumption were 1.5 and 3.6 times more likely to have the MetS respectively compared to those who reported low TV viewing time and low snack food consumption. The combination of high TV viewing time and high snack food consumption was also shown to be associated with an increased likelihood of obesity, insulin resistance and hypertension (in women only). Importantly for women, the joint association between TV viewing time and snack food consumption with the MetS and its components was not shown to be dependent on the overall quality of their diet or by the presence of central adiposity.

These are the first findings reporting on the joint association of two distinct yet modifiable lifestyle behaviors– consumption of energy-dense, nutrient poor snack foods and TV viewing – in relation to risk for the MetS and its individual components. Despite evidence to show that these two behaviors are positively correlated with one another in adults [14], previous studies have reported only on the association between TV viewing time and the MetS [3] or continuous risk scores for the MetS [6] adjusting for diet quality, which is a measure of adherence to the dietary guidelines.

Snack food consumption during TV viewing time has been postulated as a potential mechanism through which TV viewing time increases the likelihood of the MetS. In our study, high snack food consumption (women only) and high TV viewing time were also shown to increase MetS risk independent of one another, further indicating that a prudent approach would be to target both of these behaviors within population strategies aimed at reducing MetS risk in adults, particularly for women. Our observation that daily snack food consumption did not significantly attenuate the association between TV viewing time and MetS risk in men and women supports a recent study of 4511 Danish adults that found no mediating effect of snacking habits (defined as grams per day of snack foods) on the association between leisure-time sedentary behavior (which included TV time) and individual cardio-metabolic biomarkers [31]. Two previous studies have examined the impact of snack food consumption on the association between TV viewing time and obesity risk and found that consumption of food and beverage during TV viewing does partially mediate the association between TV viewing time and abdominal [11] and central obesity [14]. It is plausible that the reason why we did not observe any significant attenuation of the relationship between TV viewing time and MetS risk is because we did not adjust for snack foods directly consumed during TV viewing time; we adjusted for total serves of snack food which may have been consumed at any time across the day.

Our observation that high snack food consumption in the presence of high TV viewing exacerbated risk for the MetS and its components of obesity, insulin resistance and hypertension to a greater extent in women is supportive of previous observational studies [32,33]. It has been suggested that this may be attributed to TV viewing time being a more robust marker of sedentary behavior in women [34]. Another possible reason is that TV viewing provides a stronger stimulus for unhealthy eating behavior in women than in men. Indeed, Gore *et al.* observed the more women snack whilst watching TV the less likely they are to select low-caloric, low fat snack foods [35]. Additionally, there is evidence that TV viewing can both impair food intake recall and promote over-consumption during a later meal in women [36].

One proposed underlying mechanism for the significant joint association between TV viewing and snack food consumption with the MetS observed in our study is that watching TV simulates “mindless eating” [37]. Adults learn while watching TV to ignore internal satiety signals that trigger a feeling of “fullness” and rely on external cues, such as the conclusion of a TV program, to signal normal meal satiation [37]. Excessive energy intake from “mindless eating” coupled with a loss of localised muscular contraction induced through sitting during TV viewing may subsequently lead to transient exaggerated elevations in blood glucose, free fatty acids and triglycerides [38]. Such postprandial changes, when repeated multiple times each day, could potentially trigger pathogenic pathways known to be involved in the development of the MetS; including abnormal glucose and fat metabolism, insulin resistance and inflammation [39].

In our study, approximately one fifth of women (18.1%) and one third of men (30.8%) consumed the equivalent of more than three snack foods per day, a level which exceeds the recommended daily intake according to AGHE guidelines [24]. For women, the consumption of more than three snack foods a day was found to significantly increase risk for the MetS (94% increase) and hypertension (43% increase). This finding extends upon earlier observations that show a detrimental association between diet quality [2] and indicators of diet quality (total dietary fat intake) [40] with the MetS and identifies for the first time that high snack food consumption (reported as serves per day) is an unfavourable dietary behavior that increases MetS risk in women, independent of TV viewing time and overall diet quality. Given that adults with or at risk of having the MetS are prone to under-report their intake of energy-dense snack foods in FFQ’s [41], it is plausible that individuals with the MetS and its components in our study may have under-reported their intake of snack foods (serves per day), resulting in an underestimation of the strength of association between snacking behavior and MetS risk; further research is needed to confirm these findings.

Important strengths of the study include its large sample size and the inclusion of adults of a wide age range and without previous history of cardiovascular disease and diabetes. Reliable and valid self-administered questionnaires were also used to obtain information on lifestyle behavior and objective data was collected for all components of the WHO definition of the MetS. Our study also had a number of limitations that should be considered. Despite an acceptable response rate at baseline (55%) and weighting the sample to the estimated 1998 residential Australian population, there was an overrepresentation of higher socio-economic groups and underrepresentation of indigenous and rural populations in our study sample. This would suggest our results, while corrected for gender and age bias, are unlikely to be generalizable to the whole Australian population. Another limitation to the generalisability of our findings is that it was necessary for us to exclude a number of participants for whom only incomplete and/or invalid data on TV viewing time and FFQ variables were available.

Other limitations include the inability to differentiate from the FFQ whether participant’s self- reported intake of snack foods was consumed during TV viewing. This precluded inferences about whether the elevation in MetS risk observed in adults who reported both high TV viewing time and high snack food consumption was a direct consequence of the two behaviors occurring concurrently. Furthermore, the FFQ is limited to the number of snack foods that it can capture (n = 9) and several popular snack products are not included. Specifically, the FFQ did not capture sugar-sweetened beverage consumption, which has been shown to be independently associated with the prevalence of the MetS in adults [42].

## Conclusion

We found that daily TV viewing time and snack food consumption were jointly associated with the odds of having the MetS and its components in Australian adults without a history of cardiovascular disease or diabetes mellitus. TV viewing time and snack food consumption (in women only) were also found to be independently associated with an increased risk for the MetS and its individual components. Interpretation of our results would suggest in addition to the promotion of regular physical activity, population strategies addressing the MetS should give consideration to reducing time spent watching TV, as well as limiting excessive snack food consumption, particularly in women.

## Abbreviations

TV: Television; MetS: Metabolic syndrome; FFQ: Food frequency questionnaire; AGHE: Australian Guidelines to Healthy Eating.

## Competing interests

The authors declare that they have no competing interests.

## Authors’ contributions

AAT, SAM, NO and DWD designed research; AAT and SAM conducted research; SAM provided essential materials; AAT analysed data and performed statistical analyses; AAT, SAM, NO and DWD wrote paper; AAT had primary responsibility for final content. All authors read and approved the final manuscript.
